# Partner responses to pain among male partners of women with provoked vestibulodynia—a cross-sectional study

**DOI:** 10.1097/PR9.0000000000001265

**Published:** 2025-03-17

**Authors:** Linn Myrtveit-Stensrud, Elin Ekholm, Ida Flink, Moniek ter Kuile, Linnéa Engman, Isabelle Suvaal, Karen Synne Groven, Silje Endresen Reme

**Affiliations:** a Department of Rehabilitation Science and Health Technology, Oslo Metropolitan University, Oslo, Norway; b School of Behavioral, Social and Legal Sciences, University of Örebro, Örebro, Sweden; c Department of Social and Psychological Studies, University of Karlstad, Karlstad, Sweden; d Department of Obstetrics and Gynecology, Leiden University Medical Center, Leiden, the Netherlands; e Department of Neuroscience, Karolinska Institute, Solna, Sweden; f Faculty of Health Studies, VID Specialized University, Oslo, Norway; g Department of Psychology, University of Oslo, Oslo, Norway

**Keywords:** Vulvodynia, PVD, Vulvovaginal pain, Genito-pelvic pain, Chronic pain, Male partners, Heterosexual couples, Cross-sectional study, Partner responses

## Abstract

**Introduction:**

Provoked vestibulodynia (PVD) is a prevalent persistent pain disorder, localized to the vulva, not only affecting the sexual health of women suffering from it but also of their partners. Partner's behavioral response to the pain has implications for the affected woman's pain and sexual function, as well as the relational dynamics of the couple.

**Objectives:**

The main aim of this study was to explore facilitative, solicitous, and negative male partner responses to women with PVD and their links to demographic and psychosexual characteristics. Further, we investigated the discrepancies in psychosexual health between currently sexually active and inactive participants, as well as levels of anxiety and depressive symptoms within our sample of male partners (N = 127).

**Methods:**

Cross-sectional associations were examined using bivariate correlations. Differences in psychosexual health between the two sub-samples were examined using Mann-Whitney *U* test.

**Results:**

Our results showed that facilitative partner responses were significantly associated with higher relationship and sexual satisfaction, as well as with lower sexual distress and more approach goals. Negative partner responses were significantly associated with higher sexual distress, as well as with lower relationship and sexual satisfaction. Negative partner responses were the only responses significantly associated with the men's anxiety and depression, which suggests that mental health plays a role in partners' management of vulvodynia. In our sample, 10.2% scored above cut-off for clinical levels of depressive symptoms and 8.7% for anxiety, similar to a Swedish community sample.

**Conclusion:**

Partner responses were associated with psychosexual health. In our sample, male partners of women with PVD did not have more anxiety or depression than other men of the same age in the general population.

## Abstract 2

Supplemental Digital Content is Available in the Text.

A cross-sectional study of partner responses to pain among male partners of women with provoked vestibulodynia and their associations with psychosexual health.

## 1. Introduction

Provoked vestibulodynia (PVD), a persistent genital pain disorder, is estimated to affect between 7% and 28% of women during their lifetime.^4,29^ Provoked vestibulodynia negatively affects the quality of life of both women^2^ and their partners.^27^ Despite increasing research focus on women with PVD, there remains a significant gap when it comes to understanding how PVD affects their partners.

Qualitative studies have highlighted the communication difficulties experienced by heterosexual couples dealing with PVD, with many men struggling to understand and support their partners.^24^ Considering that the pain often occurs during sexual activity, with the partners as primary witnesses to these women's suffering,^6^ they play a critical role in the management of PVD.

Quantitative studies on male partners of women with PVD come to different conclusions regarding their mental and sexual health. Some studies find decreased sexual satisfaction, erectile function, and emotional attachment compared to controls,^26,41^ while others find no significant differences between these partners and controls.^10^

Research has indicated that partners' responses to women's pain have a significant impact on both the severity of the pain experienced and the daily functioning of patients with persistent pain [for an overview, see Refs. 28, 30]. Partner responses can be categorized as facilitative, solicitous, or negative.^30^ Facilitative responses, such as affection and encouragement, are associated with less pain, improved sexual function, and increased relationship satisfaction for both women with PVD and their partners.^31,33,35^ In contrast, solicitous and negative responses are associated with increased pain, depression, and lower sexual and relationship satisfaction.^10,31,32,34–36^ It is suggested that facilitative responses promote emotion regulation, which improves women's pain coping, while solicitous and negative responses disrupt this emotion regulation, potentially escalating fear-avoidance and perceived threat of pain.^30^ Still, there is little knowledge about which specific partner characteristics are linked to different partner responses.

Women with PVD show different coping patterns, which in turn are linked to various psychosexual outcomes, including sexual satisfaction and function.^13^ Avoidance coping is associated with pain catastrophizing,^14^ while relational catastrophizing is associated with both avoidance and endurance coping.^11^ Women who report high levels of both avoidance and endurance also report significantly higher levels of negative partner responses from their partners than those who show little avoidance and endurance coping.^11^

Enhancing our understanding of partners' experiences and behaviors is important for improving relationship satisfaction, psychosexual health, and fertility in couples dealing with PVD. Consequently, this study explores partner responses (negative, solicitous, and facilitative) among male partners of women with PVD by investigating their correlation with demographic and psychosexual characteristics. Further, given the differences in partner responses and psychosexual factors between couples with high and low levels of avoidance coping,^11,13^ we investigated the discrepancies in psychosexual health between our currently sexually active and inactive participants. In addition, considering the uncertainties surrounding the mental health of this particular group of men, we also examined levels of anxiety and depression within our sample.

## 2. Methods

### 2.1. Design and procedure

In this study, we utilized baseline data from an ongoing registered multicenter randomized waitlist-controlled trial (National Library of Medicine, NCT03427255), examining a cognitive behavioral group treatment of PVD with partner involvement. The study was approved by the Regional Board of Ethics in Uppsala (Dnr 2017/289) and by the Medical Ethics Committee Leiden-Den Haag-Delft (P17.231).

Inclusion criteria included mixed-sex couples in a stable sexual relationship of at least 3 months, where the woman was 18 to 45 years old with PVD > 6 months, with pain during at least 80% of intercourse attempts. Both partners were required to have internet access and speak Swedish or Dutch well enough for completing study questionnaires. Exclusion criteria for women included only deep dyspareunia, ongoing pregnancy or being <1 year postpartum, never having had vaginal intercourse, no intercourse with their partner in the past year, concurrent psychological treatment or pelvic floor therapy for PVD, severe psychiatric disorders such as major depression, or post-traumatic stress disorder related to the genitals after abuse, according to the Diagnostic and Statistical Manual of Mental Disorders, Fifth Edition criteria.^1^ In addition, couples were excluded if they planned to be apart for more than 3 weeks during the study period.

Couples were recruited through outpatient clinics treating women with PVD in Sweden and the Netherlands, and through free and paid advertisements on social media. Potential female participants were provided with detailed information about the study via email, followed by a phone screening of the women conducted by a research team member. Those not excluded during the screening process were then scheduled for an in-person baseline assessment with their partners, involving a semi-structured interview, baseline questionnaires, and a physical examination of the Dutch sample's female participants. In Sweden, women were instructed to consult a gynecologist for an examination and formal diagnosis before starting treatment. All participants read and signed informed consents before or at the beginning of the in-person assessment. Due to the COVID-19 pandemic, this procedure was conducted online in 2020, leading to some gynecological examinations being postponed and replaced with details from participants' medical records.

### 2.2. Participants

**Figure 1.**
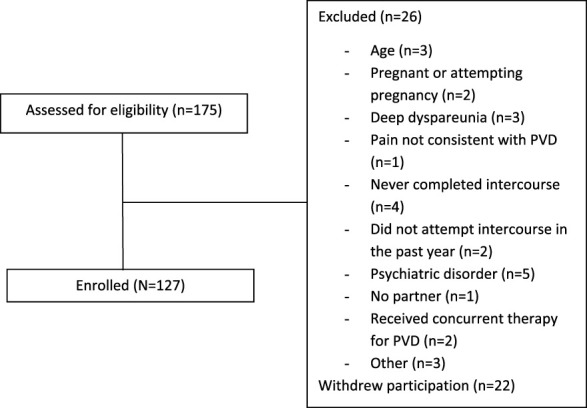
Flowchart of inclusion and exclusion of couples.

In the current study, only data from the male partners were used, in total 60 Swedish and 67 Dutch men. For an overview of the procedure for inclusion, and reasons for exclusion, see Figure 1.

### 2.3. Measures

All variables were measured through self-report questionnaires, in Swedish or Dutch, depending on location. In case Swedish or Dutch versions of the questionnaires were lacking, a translation-back-translation procedure was used. None of the new translated measures are validated for this population in Sweden or the Netherlands.

*Demographic information* included self-reported age, nationality, educational level, relationship duration, number of children, and cohabitation status. Education level was measured at 6 levels (elementary/secondary/high school, vocational training/bachelor's degree, or master's degree/PhD).

*Sexual satisfaction* was measured with the 5-item Global Measure of Sexual Satisfaction (GMSEX),^21^ where responders rate their sexual relationship (eg, pleasant or unpleasant) from 1 to 7. The total score ranges from 5 at worst to 35 at best. Internal consistency in the current study was good, with α = 0.91.

*Sexual distress* was measured with the Female Sexual Distress Scale (FSDS),^9^ where 12 items (eg, “guilty about sexual problems”) were scored on how frequently they caused distress over the past 30 days (0 = Never, to 4 = Always). Since the FSDS does not contain gender-specific items, the measure has also been successfully administered to male participants.^15,18^ It has shown good content, construct, and criterion validity, as well as good internal consistency reliability and test–retest reliability in previous validation studies for assessing sexual distress in male samples.^39^ The total score ranges from 0 at best to 48 at worst distress. Internal consistency in the current study was good with α = 0.90.

*Interpersonal sexual motives and goals* were measured using Approach and Avoidance Sexual Goals,^7,23,37^ asking participants to rate the importance of each item for engaging in sex on a scale from 1 (=Not at all important) to 7 (=Extremely important). The Approach subscale consists of 10 items (eg, “To experience pleasure with my partner”), and the Avoidance subscale comprises 6 items (eg, “To avoid conflict in my relationship”). The total score ranges from 10 to 70 for the approach subscale, and from 6 to 42 for the avoidance subscale. Internal consistency of the 2 subscales was good, with α = 0.84 for the approach subscale and α = 0.89 for the avoidance subscale.

*Partner (behavioral) responses* were assessed by MPI-SR, including 16 of the original 20 items of the questionnaire,^33^ which was adapted to couples experiencing pain in sexual situations. This questionnaire is based on the West Haven-Yale Multidimensional Pain Inventory Significant Other Response Scale (MPI)^19^ and the Spouse Response Inventory Facilitative subscale (SRI).^40^ There are 2 versions, 1 for the person in pain, and 1 for their responding partners, with the last 1 answered by the participants of our study. The MPI-SR consists of 3 subscales representing different partner responses to pain. The subscale Solicitous consists of 6 items (eg, “I suggest we turn on the T.V. or sleep”), the Negative subscale of 4 items (eg, “I express anger at her”), and the Facilitative subscale comprises 6 items (eg, “I say nice things to her”). Respondents are asked to rate each item on a scale from 1 (=“Never”) to 6 (=“Very frequently”). The total score ranges from 6 to 36 for the solicitous and facilitative subscales, and from 4 to 24 for the negative subscale. Internal consistency for the subscales was good for the negative and facilitative with α = 0.82 and 0.78, respectively. Internal consistency was lower for the solicitous subscale, with α = 0.64.

*Male sexual function* was assessed with the 15 items of the International Index of Erectile Function (IIEF),^38^ which is a widely used, multidimensional self-report instrument for the evaluation of male sexual function. The total score ranges from 5 at worst to 75 at best. Internal consistency for the IIEF was good with α = 0.92.

*Relationship dissatisfaction* is assessed with the 10 items of the marital dissatisfaction subscale of the Maudsley Marital Questionnaire (MMQ).^3,5,8^ The total score ranges from 0 at best to 80 at worst dissatisfaction. Internal consistency for the MMQ was good, with α = 0.81.

*Mental health* was assessed by depression and anxiety symptoms on the 9 item Patient Health Questionnaire (PHQ-9)^20^ and 7 item generalized anxiety disorder-7 (GAD-7).^42^ We have used a cut-off score for assessing clinical levels of anxiety and depression symptoms of ≥8 (GAD) and ≥10 (PHQ), respectively. The total score ranges from 0 at best to 27 at worst depression, and from 0 at best to 21 at worst anxiety. Internal consistency for the PHQ-9 and GAD-7 was good, with α = 0.83 and α = 0.78, respectively.

### 2.4. Data analyses

The analyses were conducted using IBM Statistical Package of Social Sciences (SPSS) 29. Initially, the data were summarized and examined using descriptive and correlational statistics (Table 1).

**Table 1:** Descriptive results for the sample at baseline (N = 127).

Age (y)	
Median (range)	28.0 (20–52)
Nationality	
Swedish	58 (46%)
Dutch	64 (50%)
Other	5 (4%)
Relationships length (y)	
Median (range)	4.3 (0.7–16.8)
Living situation	
Cohabiting	94 (74%)
Living apart	33 (26%)
Level of education	
Elementary/secondary school	7 (5.5%)
High school	37 (29.1%)
Vocational education	25 (19.7%)
Bachelor's degree	30 (23.6%)
Master's degree or PhD	27 (21.3%)
Unknown	1 (0.8%)
Anxiety	
Score range	0–21
Mean (SD)	3.5 (3)
Median (range)	3 (0–19)
Above cut-off (≥8)	11 (8.7%)
Depressive symptoms	
Score range	0–27
Mean (SD)	4.3 (4)
Median (range)	4 (0–25)
Above cut-off (≥10)	13 (10.2%)
Sexual function	
Score range	5–75
Mean (SD)	34.1 (18.9)
Median (range)	30 (9–73)
Sexual distress	
Score range	0–48
Mean (SD)	14.2 (13.6)
Median (range)	13.5 (0–36)
Sexual satisfaction	
Score range	5–35
Mean (SD)	24.3 (7.1)
Median (range)	25 (10–35)
Relationship dissatisfaction	
Score range	0–80
Mean (SD)	10 (7.2)
Median (range)	9 (0–39)
Approach sexual goals	
Score range	10–70
Mean (SD)	50.4 (10.3)
Median (range)	51 (24–69)
Avoidance sexual goals	
Score range	6–42
Mean (SD)	18.6 (9.4)
Median (range)	17 (6–42)
Sexual activity	
Intercourse last 4 wk	77 (60.6%)
No intercourse last 4 wk	50 (39.4%)

First, the internal consistency of all the multi-item measures was examined, that is, the extent to which all the items measure the same concept or construct. Cronbach alpha was used. In the case of the partner response questionnaire (MPI-SR) and the approach and avoidance sexual goals, alpha was calculated for each concept (latent variables) as opposed to the entire scale.

Second, we investigated differences between 2 subgroups of our sample: men who reported being currently sexually active and those who reportedly were not (Table 2). Mann–Whitney *U* test was used.

**Table 2:** Differences between sexually active (n = 77) and inactive (n = 50) participants.

	Sexually active participants (N = 77)	Sexually inactive participants (N = 50)	Mann–Whitney U	Standardized test statistic (z score)	*P*
Anxiety (GAD-7)			1888.00	−0.184	0.854
Mean (SD)	3.3 (2.6)	3.7 (3.6)			
Median (range)	3 (0–9)	3 (0–19)			
Depressive symptoms (PHQ-9)			1854.00	−0.164	0.870
Mean (SD)	4.1 (3.4)	4.7 (4.8)			
Median (range)	4 (0–17)	4 (0–25)			
Sexual function (IIEF)			3043.50	5.977	<0.001**
Mean (SD)	42.2 (18.4)	21.6 (11.6)			
Median (range)	43.5 (17–73)	15 (9–44)			
Sexual distress (FSDS)			1270.50	−3.142	0.002**
Mean (SD)	12.73 (7.8)	17.1 (8.3)			
Median (range)	11 (0–31)	17.5 (0–36)			
Sexual satisfaction (GMSEX)			2539.50	4.186	<0.001**
Mean (SD)	26.5 (6.4)	20.8 (7)			
Median (range)	28 (11–35)	18 (10–35)			
Relationship dissatisfaction (MMQ)			1575.00	−1.730	0.084
Mean (SD)	8.8 (6.4)	12 (8.8)			
Median (range)	9 (0–22)	9 (0–39)			
Approach sexual goals (AASQ)			1869.00	−0.277	0.782
Mean (SD)	50 (10.5)	50.8 (10.1)			
Median (range)	51 (24–69)	51 (26–69)			
Avoidance sexual goals (AASQ)			1902.00	−0.114	0.910
Mean (SD)	18.4 (9.3)	18.8 (9.7)			
Median (range)	17 (6–42)	18 (6–40)			

Values were rounded up to 2 decimals. Bold values indicate statistically significant findings.

*P < 0.05, **P < 0.01.

Third, an explorative, bivariate correlation analysis was conducted to investigate which measures (Table 3) can explain the variation in the 3 responses (facilitative, solicitous, negative). Spearman rank correlation coefficient (rho) was used. With almost 40% of our participants reporting no intercourse in the last 4 weeks, we considered their responses to the MPI-SR might not be valid, since the MPI-SR asks about partner responses to pain during intercourse. Therefore, the correlational analyses were conducted for the currently sexually active subsample only (n = 77). Moreover, we performed a sensitivity analysis using the complete sample (N = 127) to test the robustness of our findings, including both those who reported having had sexual intercourse in the past 4 weeks and those who reported that they had not (Supplementary Table 1, http://links.lww.com/PR9/A297). While this analysis revealed some discrepancies, the general trends in the data largely remained the same.

**Table 3:** Bivariate correlations for the currently sexually active sample (n = 77).

	Facilitative responses	Solicitous responses	Negative responses
Sexual function (IIEF)	−0.04	−0.16	−0.10
Sexual satisfaction (GMSEX)	0.34**	−0.09	−0.47**
Sexual distress (FSDS)	−0.27*	0.05	0.35**
Relationship dissatisfaction (MMQ)	−0.40**	−0.10	0.39**
Depressive symptoms (PHQ-9)	−0.07	0.05	0.26*
Anxiety (GAD-7)	−0.07	0.02	0.28*
Approach goals	0.35*	0.07	−0.10
Avoidance goals	0.10	−0.06	−0.07
Facilitative responses		0.46**	−0.15
Solicitous responses	0.46**		0.01
Negative responses	−0.15	0.01	
Age	−0.04	0.06	0.04
Relationship length	−0.15	−0.01	0.18

Values were rounded up to 2 decimals. Bold values indicate statistically significant findings.

*P < 0.05, **P < 0.01.

## 3. Results

### 3.1. Sample descriptives

As shown in Table 1, the median age of our 127 participants was 28 years, ranging from 20 to 52 years. The median length of their romantic relationship was 4.3 years, ranging from less than a year to more than 16 years. Half of the participants were Dutch, while 46% were Swedish, and a few were from other countries (4%). Most of the participants were cohabiting (74%). Furthermore, Table 1 provides the educational distribution, with the highest percentage being high school graduates (29.1%). The levels of clinical anxiety and depressive symptoms were 8.7% and 10.2%, respectively, based on suggested cut-off scores by Johansson et al.^17^ When asked about attempts at sexual intercourse in the last 4 weeks, almost 40% of the participants reported none.

### 3.2. Differences between currently sexually active and inactive participants

As can be seen in Table 2, the results of the Mann–Whitney *U* test revealed significantly lower sexual satisfaction and higher sexual distress among men who reported no intercourse in the last 4 weeks (ie, inactive) compared to sexually active participants. Furthermore, there were no significant differences between these 2 groups in mental health outcomes or relationship satisfaction.

### 3.3. Partner responses to the woman's pain

To address validity concerns surrounding partner responses to intercourse pain in currently sexually inactive participants, we limited the analyses of partner responses shown in Table 3 to sexually active participants only. The participants reported their responses to their female partner's pain during sexual activity, and the responses were divided into 3 subscales for facilitative, solicitous, and negative partner responses. The distribution of negative responses was markedly skewed, with most participants reporting few negative responses compared to facilitative and solicitous responses. With a significance level of *P* < 0.01, our results show significant positive associations between facilitative responses and higher sexual satisfaction, as well as lower relationship dissatisfaction (ie, higher satisfaction). Negative partner responses were significantly associated with higher sexual distress and relationship dissatisfaction, as well as lower sexual satisfaction. Our results also show a significant positive association between facilitative and solicitous partner responses (r = 0.46), suggesting that these subscales measure similar behaviors. With a significance level of *P* < 0.05, facilitative responses were also associated with lower sexual distress and more approach goals, while negative responses were associated with higher reported levels of anxiety and depressive symptoms.

As a sensitivity analysis, we conducted the analysis with the full sample, including participants who reported no sexual intercourse in the last 4 weeks. While the results of this analysis revealed some deviations, the overall correlations remained largely consistent. In addition to the significant results found with the subsample, the full sample also showed significant positive correlations between solicitous responses and sexual distress (*P* < 0.05), as well as negative responses and depressive symptoms (*P* < 0.05).

## 4. Discussion

This study explored partner responses to pain among male partners of women with PVD and their links to demographic and psychosexual characteristics. Further, we investigated the discrepancies in psychosexual health between our currently sexually active and inactive participants, as well as levels of anxiety and depressive symptoms within our sample.

The men reported more facilitative and solicitous than negative responses, suggesting that they want to be considerate of their partner's pain. The combination of these response styles might be due to the questionnaire not being able to distinguish well between the 2, which is supported by the significant association between these 2 subscales. However, these results can also indicate that the men trying to be supportive express a combination of these responses, as they are both attempts at conveying support, although with different consequences.

In line with previous research, our results show that facilitative partner responses are significantly associated with higher relationship and sexual satisfaction, as well as with lower sexual distress and more approach goals. Ideally, most pain expressions from the woman should be met with facilitative responses, as previous research has shown that partners who validate their partner's pain report greater satisfaction with their relationship, while partners who are less satisfied and experience pain themselves validate the least.^16^ At the same time, determining the ideal response in a particular situation is challenging, and what might be considered facilitative in 1 situation might be solicitous in another. For instance, one of the items of the MPI-SR measurement is *“I suggest we stop engaging in current sexual activity*.”^33^ This action might be understood as solicitous because it encourages the women to avoid sexual activity, but it can also be understood as facilitative by avoiding a painful activity to find better alternatives. Sometimes facilitative responses require cooperation from the partner, eg, honesty about pain during different activities. If the men do not trust that their partner tells them about their pain, which qualitative studies have suggested is the case,^25^ it is also difficult to facilitate constructive solutions. In those circumstances, it might be considered easier to respond solicitously to avoid further possible pain and guilt. Solicitous responses could also be a means of avoiding sexual activity due to fear of rejection, as observed in qualitative research.^25^ In addition, it is possible that some men in our study refrained from sexual activity due to concerns about their sexual performance, rather than the sexual function of their partner. While these 2 kinds of responses might seem similar, facilitative responses are associated with more positive outcomes for women than solicitous responses.^30^

Our sample shows a relatively low prevalence of negative partner responses compared to more positive responses. This might be due to self-reporting and social desirability, or a potential selection bias of participants to a treatment study, or that the men are mostly supportive of their partners. However, negative partner responses were significantly associated with higher sexual distress, as well as with lower relationship and sexual satisfaction. Negative partner responses were the only responses significantly associated with anxiety and depressive symptoms, which suggests that the men's mental health plays a role in their management of vulvodynia. Previous research shows that male partners of women with vulvodynia may react with considerable shame and guilt when they see their partner in pain,^24^ perhaps heightened by anxiety and depression. These feelings might lead to less constructive responses due to their own negative emotions, rather than a lack of sympathy for their partner.

Prior research has indicated that couples respond to vulvodynia in various ways. Some opt to completely avoid any sexual activity, while others may endure it despite the woman's pain.^11^ Others find more constructive ways to alter their shared sexuality, aiming to reduce pain and enhance pleasure. In our sample, almost 40% of the men reported no attempts at intercourse in the last 4 weeks, with some of these likely being in relationships with women with high avoidance coping. Interestingly, there were no significant differences between our 2 subgroups in mental health outcomes or relationship satisfaction. These findings suggest that vulvodynia may primarily influence the sexual aspect of a relationship, while not significantly affecting the overall quality of the relationship or the individuals' mental well-being.

Sexual function, as assessed by the International Index of Erectile Function, was predictably lower in the group abstaining from intercourse, due to its emphasis on intercourse frequency. This metric may reflect an underrating of sexual function among these men, who may refrain from intercourse primarily due to their partner's sexual issues, not their own. Conversely, scores for sexual distress (FSDS) and satisfaction (GMSEX)—which consider a broad spectrum of sexual activities—also exhibit notable discrepancies across groups engaging in varying levels of intercourse. Our findings suggest that the currently sexually inactive, despite the potential for engaging in noncoital sexual activities that could enhance satisfaction, report greater sexual distress, and lower satisfaction. These outcomes may be influenced by their partners' pain and coping strategies, an area warranting further exploration. However, it remains unclear whether dissatisfaction and distress among these men stem from low sexual activity or if their inactivity is a consequence of these negative feelings.

Previous studies show somewhat contrasting results related to the mental health of male partners of women with PVD. One study found a significant difference in depressive symptoms, but not anxiety, between partners of women with vulvodynia and controls,^26^ while another study found no significant differences in mental health,^10^ and 1 study even found that partners scored significantly lower on measures of psychopathology than a normal population.^43^ As recommended by a meta-analysis of the depression scale PHQ-9,^22^ we used a cut-off score of 10 or above to determine the rate of clinical depressive symptoms in our sample. This gave us a prevalence of 10.2% with clinical depressive symptoms in our sample, slightly higher than the 8.3% found in a Swedish community sample.^17^ Regarding the anxiety measure (GAD-7), a cut-off point of 10 or greater is recommended by some studies,^42^ while the previously mentioned Swedish community study^17^ used a cut-off score of ≥8. For the purpose of comparison, we also used a cut-off of ≥8, giving a clinical anxiety prevalence of 8.7%, which is lower than the 10.7% found in the Swedish community sample.^17^ These results indicate that our sample presents a slightly higher frequency of men with clinically depressive symptoms and a lower frequency of clinical anxiety compared to a Swedish community sample. However, our results do not suggest that male partners of women with PVD have worse mental health than other men of the same age in the general population.

The key strengths of this study are rooted in its multisite clinical sample, with recruitment from different contexts and countries enhancing the external validity of the findings. However, the results might differ from cultures with less gender equality or differently organized health care.

A limitation of our study is its cross-sectional design, which precludes conclusions regarding the direction of the associations or how these associations might affect psychosexual outcomes over time. In addition, the results are self-reported, which might influence the reported partner responses, as negative responses are less desirable to report than the other responses. Future studies could perhaps get more reliable reports by observing couples or combining data from both members of a couple. Another limitation is the sample size, especially after the original sample of 127 was divided into 2 subsamples for analysis. To address this limitation, we conducted a sensitivity analysis by rerunning the analysis with the full sample. There is also uncertainty surrounding the 22 couples that withdrew from the study, although some indicated the treatment's extensive time commitment as their reason for withdrawal. It is unclear how these couples may have differed from those included in the study.

This patient group likely underrepresents individuals from ethnic minorities or those with low socioeconomic status, possibly due to less effective dissemination of study information to these communities compared to the majority population. There is also a bias in who gets diagnosed with PVD, as it typically takes significant personal and economic resources for women to achieve the right diagnosis and treatment, as well as relatively high health literacy.

Previous research reveals recruitment challenges, possibly because men view vulvodynia as solely their partner's issue.^24^ Moreover, as many women do not discuss their pain with their partners,^12^ these men are less likely to be aware of or involved in research studies on the condition.

The study's requirement for physical attendance may bias participation towards cohabiting couples, likely explaining their high representation. Most participants were in their 20s, indicating that our results might not be generalizable to older couples. Despite this selectivity, our participants likely mirror many couples encountered in clinical settings by health professionals, as these couples typically have discussed the disorder and are invested in improving the women's treatment outcomes.

In conclusion, our results corroborate previous research, highlighting the associations between facilitative partner responses and positive relationship outcomes. Meanwhile, negative responses are linked to more detrimental outcomes. Contrary to some prior studies, our results indicated similar rates of anxiety and depressive symptoms compared to the general population. Based on our findings, we propose that the inclusion of both partners in the treatment of PVD may be beneficial for improving relational dynamics, especially related to partner responses to pain.

## Disclosures

The authors report there are no competing interests to declare.

## Appendix A. Supplemental digital content

Supplemental digital content associated with this article can be found online at http://links.lww.com/PR9/A297.

## Supplementary Material

SUPPLEMENTARY MATERIAL
