# Supplementary material for: Partner responses to pain among male partners of women with provoked vestibulodynia—a cross-sectional study
Source: Pain Rep. 2025 Mar 17;10(2):e1265. doi: 10.1097/PR9.0000000000001265 (PMC11918749; doi:10.1097/PR9.0000000000001265)
Supplement: SUPPLEMENTARY MATERIAL [file painreports-10-e1265-s001.pdf]

Supplementary table 1

Bivariate correlations for the complete sample (N=127).

	**Facilitative responses**	**Solicitous responses**	**Negative responses**
Sexual function (IIEF)	.05	-.16	-.06
Sexual satisfaction (GMSEX)	**.35****	-.16	**-.34****
Sexual distress (FSDS)	**-.30****	**.19***	**.38****
Relationship dissatisfaction (MMQ)	**-.42****	.01	**.31****
Depressive symptoms (PHQ-9)	.00	.07	.15
Anxiety (GAD-7)	-.04	.12	**.23***
Approach goals	**.24****	.10	.02
Avoidance goals	.04	.03	.05
Facilitative responses		**.34****	-.07
Solicitous responses	**.34****		.11
Negative responses	-.07	.11	
Age	-.15	.00	.05
Relationship length	-.17	.00	.15

Note: *p<.05, **p<.01. Values were rounded up to two decimals.
